# Image‐guided mathematical modeling for pharmacological evaluation of nanomaterials and monoclonal antibodies

**DOI:** 10.1002/wnan.1628

**Published:** 2020-04-21

**Authors:** Prashant Dogra, Joseph D. Butner, Sara Nizzero, Javier Ruiz Ramírez, Achraf Noureddine, María J. Peláez, Dalia Elganainy, Zhen Yang, Anh‐Dung Le, Shreya Goel, Hon S. Leong, Eugene J. Koay, C. Jeffrey Brinker, Vittorio Cristini, Zhihui Wang

**Affiliations:** ^1^ Mathematics in Medicine Program Houston Methodist Research Institute Houston Texas USA; ^2^ Department of Chemical and Biological Engineering University of New Mexico Albuquerque New Mexico USA; ^3^ Applied Physics Graduate Program Rice University Houston Texas USA; ^4^ Department of Radiation Oncology University of Texas MD Anderson Cancer Center Houston Texas USA; ^5^ Center for Bioenergetics Houston Methodist Research Institute Houston Texas USA; ^6^ Nanoscience and Microsystems Engineering University of New Mexico Albuquerque New Mexico USA; ^7^ Cancer Systems Imaging University of Texas MD Anderson Cancer Center Houston Texas USA; ^8^ Biological Sciences Platform, Sunnybrook Research Institute Toronto Ontario Canada; ^9^ Department of Medical Biophysics University of Toronto Toronto Ontario Canada; ^10^ Department of Chemical and Biological Engineering and UNM Comprehensive Cancer Center University of New Mexico Albuquerque New Mexico USA

**Keywords:** mathematical modeling, monoclonal antibodies, nanoparticles, noninvasive imaging, pharmacokinetics

## Abstract

While plasma concentration kinetics has traditionally been the predictor of drug pharmacological effects, it can occasionally fail to represent kinetics at the site of action, particularly for solid tumors. This is especially true in the case of delivery of therapeutic macromolecules (drug‐loaded nanomaterials or monoclonal antibodies), which can experience challenges to effective delivery due to particle size‐dependent diffusion barriers at the target site. As a result, disparity between therapeutic plasma kinetics and kinetics at the site of action may exist, highlighting the importance of target site concentration kinetics in determining the pharmacodynamic effects of macromolecular therapeutic agents. Assessment of concentration kinetics at the target site has been facilitated by non‐invasive in vivo imaging modalities. This allows for visualization and quantification of the whole‐body disposition behavior of therapeutics that is essential for a comprehensive understanding of their pharmacokinetics and pharmacodynamics. Quantitative non‐invasive imaging can also help guide the development and parameterization of mathematical models for descriptive and predictive purposes. Here, we present a review of the application of state‐of‐the‐art imaging modalities for quantitative pharmacological evaluation of therapeutic nanoparticles and monoclonal antibodies, with a focus on their integration with mathematical models, and identify challenges and opportunities.

This article is categorized under:

Therapeutic Approaches and Drug Discovery > Nanomedicine for Oncologic Disease
Diagnostic Tools > in vivo Nanodiagnostics and Imaging
Nanotechnology Approaches to Biology > Nanoscale Systems in Biology

## Graphical abstract

Pharmacological evaluation of nanoparticles and antibodies performed through quantitative imaging‐guided mathematical models.

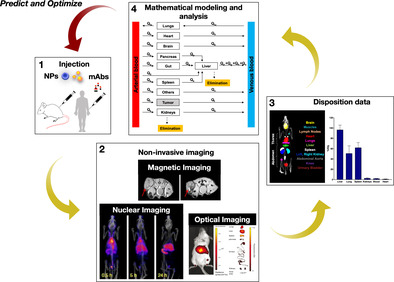

## 1 INTRODUCTION

Following drug administration into an in vivo system, the interplay between the pharmacokinetic (PK) processes of absorption, distribution, metabolism, and excretion (ADME) governs the plasma (or systemic) concentration kinetics of the drug, which is traditionally regarded as a predictor of the therapeutic efficacy or toxicity of the given drug. Plasma kinetics has been used to evaluate the pharmacological exposure‐response relationships under the assumption that plasma concentration is a surrogate for drug concentration at the site of action (Gabrielsson & Weiner, 2001). However, due to challenges associated with drug chemistry, dosage form, inter‐individual variability, or biological differences between healthy and diseased tissues, drugs may not effectively reach the site of action to achieve a therapeutic concentration locally, despite achieving a therapeutically relevant concentration systemically (Nizzero, Ziemys, & Ferrari, 2018; Rizk, Zou, Savic, & Dooley, 2017). This can result in differential therapeutic responses across treatments, or across patients, and can often lead to therapeutic resistance (T. Brocato et al., 2014), suggesting that the plasma concentration does not always reflect the target site concentration of the drug and highlighting the importance of target site concentration as the true predictor of drug effects. Further, assessment of therapeutic concentration at the target site becomes increasingly relevant when nanomaterials such as nanoparticle (NP) drug carriers or nano‐sized therapeutic monoclonal antibodies (mAbs) are involved. This is primarily due to the additional physical constraints imposed by biological barriers that impede the transport of these nano‐sized entities to the site of action due to their large particle size compared to free drugs (Dreher et al., 2006; Nizzero, Shen, Ferrari, & Corradetti, 2019; Schmidt & Wittrup, 2009; Tang et al., 2014; Thurber, Schmidt, & Wittrup, 2008; Thurber, & Weissleder, 2011), thereby creating greater disparity between plasma concentration and target site concentration for these macromolecules (*for the sake of brevity*, *wherever feasible*, *we will collectively refer to NPs and mAbs as macromolecules throughout this manuscript*).

The plasma concentration kinetics of macromolecules provides an incomplete understanding of their whole‐body distribution kinetics, as macromolecules do not achieve uniform dispersion across tissues, primarily due to their predominant distribution and prolonged retention in the liver and spleen (Dogra et al., 2018; L. Liu, 2018; Tsoi et al., 2016). It thus becomes imperative to accurately assess the concentration kinetics of macromolecules discretely at the site of action, in addition to unravelling the whole‐body kinetics for a complete understanding of their in vivo disposition. To date, efforts towards this end have been hindered by challenges associated with the assessment of therapeutic concentrations in the tissues or biological fluids of interest (Rizk et al., 2017), and plasma concentration kinetics has remained a popular choice for pharmacological evaluation of macromolecules. Advancements in non‐invasive in vivo imaging (Ding & Wu, 2012; Smith & Gambhir, 2017) are helping to overcome these limitations, and it is now becoming increasingly common to conduct whole‐body imaging over time (Dogra et al., 2018) to investigate the diseased site kinetics of NPs (Goel et al., 2019; Meng, Wang, Ping, & Yeo, 2018) and mAbs (Cole et al., 2017; Niemeijer et al., 2018), and thus obtain a comprehensive understanding of the PK behavior of macromolecules, which can explain the therapeutic benefits or insufficiencies associated with these xenobiotics (Ng, Garlin, Weissleder, & Miller, 2020).

**FIGURE 1:**
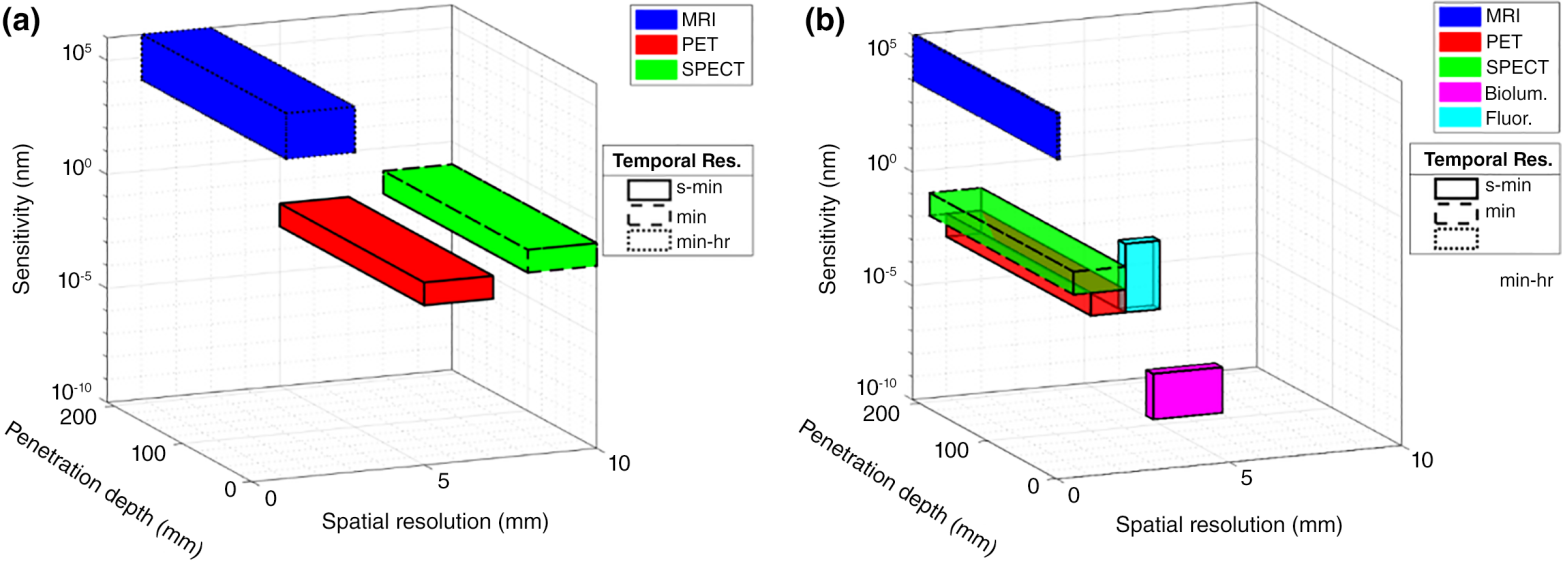
Spatiotemporal resolution, penetration depth, and sensitivity of the key imaging modalities in (a) clinical and (b) preclinical settings. Temporal resolution is represented by solid (few seconds to minutes), dashed (few minutes), and dotted (few minutes to hour) boundaries of the cuboids whose color denotes the imaging modality‐type, and the *x*‐, *y*‐, and *z*‐axis represent spatial resolution, penetration depth, and sensitivity, respectively. Key: MRI, magnetic resonance imaging; PET, positron emission tomography; SPECT, single photon emission computed tomography; biolum., bioluminescence; fluor., fluorescence

Several imaging modalities are available to quantify macromolecules at the diseased site or in healthy compartments of the body in both preclinical and clinical settings. This is typically achieved by labeling the macromolecules with imaging agents; these are generally small molecules that, upon stimulation with an input signal, or without, produce a reporter signal that is recognized by the receiver device of the imaging modality to produce a time‐varying image sequence across the body (Smith & Gambhir, 2017). The most commonly used quantitative imaging modalities for living systems that are discussed in detail in this article are magnetic imaging, nuclear imaging, and optical imaging. These modalities can vary considerably in their spatiotemporal resolution, penetration depth, and sensitivity (see Figure 1), thereby providing guidelines for the selection of the appropriate modality for a given macromolecule. The non‐invasive nature of these modalities improves patient compliance and allows for the investigation of macromolecules in an unperturbed in vivo environment. The series of images obtained through non‐invasive imaging can be quantified to guide the development of empirical or mechanistic mathematical models. The former modeling approach can be used to describe the in vivo disposition kinetics and pharmacodynamics of macromolecules, and the latter can be used as an in silico tool to investigate the role of physicochemical or pathophysiological factors in affecting the pharmacology of macromolecules, in addition to predicting their spatiotemporal concentration dynamics. The mathematical models thus developed can vary significantly in terms of the time‐ or length‐scales they represent (Dogra, Butner, et al., 2019), which are selected by the modeler based on the biological system being modeled and any limitations of the spatiotemporal resolution of the available imaging data for model parameterization and validation, among other factors.

Increased application of non‐invasive quantitative imaging will likely allow for the development of new therapeutics, improve diagnosis, and allow real‐time monitoring of treatment and its optimization. In the following, we will *i*) discuss the biophysical factors that govern the transport of macromolecules, thereby affecting their pharmacology, *ii*) present the key imaging modalities that are used in vivo or in the clinic for macromolecule visualization and estimation of model parameters, and *iii*) review key imaging‐driven mathematical models for pharmacological evaluation of macromolecules intended for therapeutic, diagnostic, or theranostic (e.g., hybridized therapeutic and diagnostic) applications in cancer.

## 2 TRANSPORT PHYSICS

**FIGURE 2:**
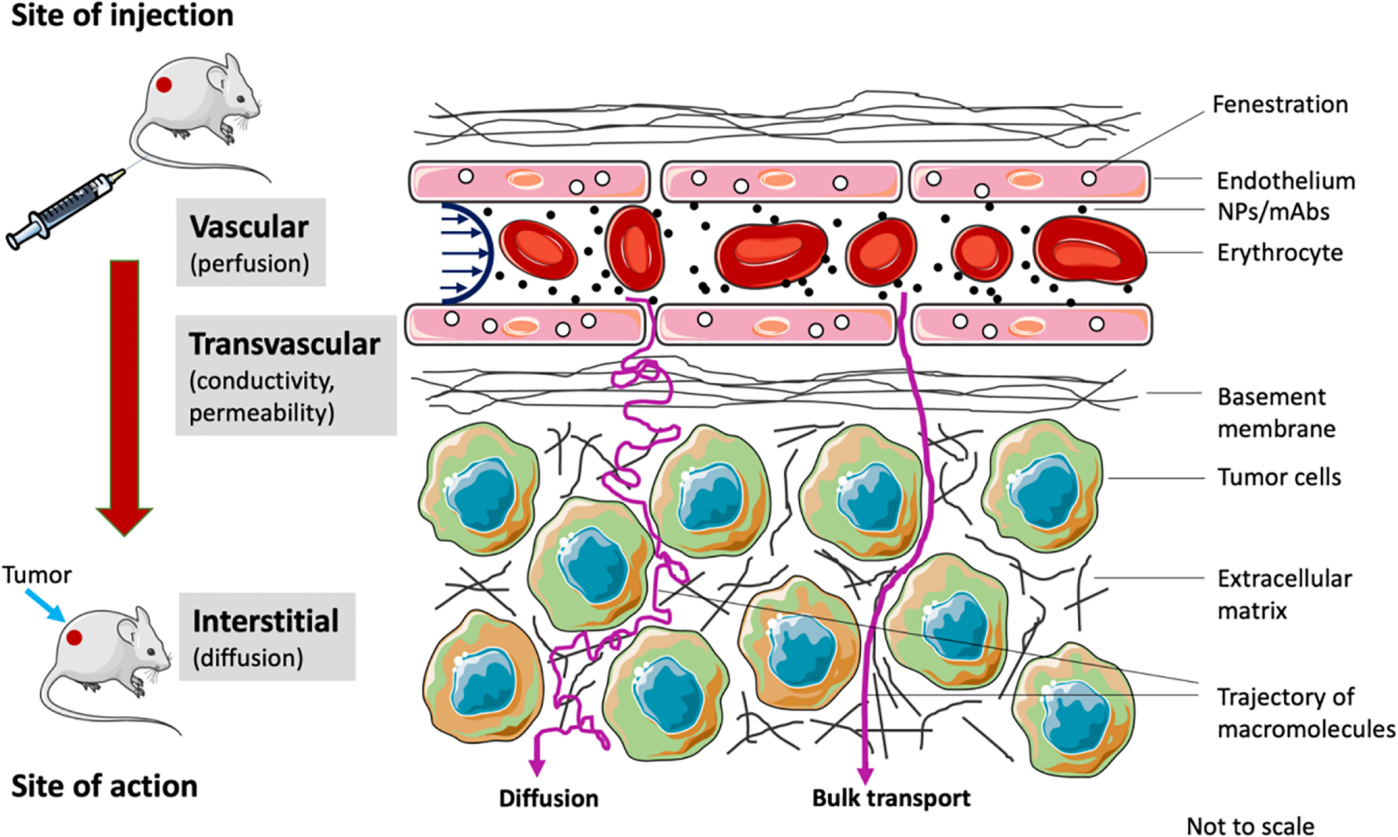
Transport of macromolecules from site of injection to target site. Delivery of nanoparticles and monoclonal antibodies to the site of action is characterized by perfusion, hydraulic conductivity, permeability, and diffusion. (Reprinted with permission from Dogra, Butner, et al. (2019). Copyright 2019 Springer)

Efficient delivery of drugs, or macromolecules, to the site of action is essential to produce the desired pharmacological effects. Delivery to the target site is dominated by the physical transport phenomena of perfusion, microvascular extravasation, and interstitial migration, which represent the key transport processes in the vascular, transvascular, and interstitial phases of delivery, respectively (see Figure 2) (R. K. Jain & Stylianopoulos, 2010). Perfusion refers to the passage of blood through the vascular network of an organ or tissue. It is quantified as the blood flow rate per unit mass of organ (units, ml/min/g), is a property of the biological system, and varies across tissues and across species (Shah & Betts, 2012). Blood flow rate *Q* can be expressed as the ratio of the pressure difference between arterial and venous ends (Δ*P*), and flow resistance (FR):

(1) Q=ΔP/FR

(R. K. Jain & Stylianopoulos, 2010). Flow resistance is a product of apparent viscosity (due to high erythrocyte density) and geometrical resistance, which tends to be greater in tumors compared to healthy tissues, due to vessel deformation and abnormalities, thus lowering perfusion. As such, the perfusion‐dependent influx rate of macromolecules into the region of interest (ROI) is defined by the product of *Q* and macromolecule concentration in the systemic circulation.

The capillary microvasculature is a highly dynamic region of the vascular system, due to its involvement in the exchange of nutrients and waste materials between the blood and interstitial space. This exchange is feasible due to certain microanatomical features of the capillaries, such as interendothelial gaps and cellular pores or fenestrations within the single endothelial cell membrane that forms the capillary wall (Sarin, 2010). These properties allow fluid filtration through the capillary wall and make it semipermeable to solutes. There are two key mechanisms through which transvascular extravasation of substances occurs: diffusion and bulk transport. Small molecules can cross the capillary wall via diffusion due to the concentration gradient across the wall. However, due to size constraints, macromolecules do not diffuse as effectively across the capillary wall, but instead primarily rely on bulk transport to extravasate into the interstitium. Bulk transport occurs because of net filtration of fluid, generally out of the capillary into the interstitium due to the difference in hydrostatic and osmotic pressures on the two sides of the capillary wall, and is the dominant mechanism of transvascular flow of macromolecules in healthy tissues.

The combined effect of diffusion and bulk transport that governs the flux *J* of macromolecules through the capillary wall into the interstitium can be expressed as (R. K. Jain & Stylianopoulos, 2010):

(2) $$J=\mathrm{PS}\left(C_{p}-C_{i}\right)+\mathrm{LS}\left(1-\sigma \right)\left[\left(p_{v}-p_{i}\right)-\sigma \left(\pi_{v}-\pi_{i}\right)\right]C_{p}.$$

where *P* represents vascular permeability, *S* is the vascular surface area, *C*_p_ is the macromolecule concentration in systemic circulation, *C*_i_ is the interstitial concentration of macromolecules, *L* is hydraulic conductivity, *σ* is the reflection coefficient, *p*_v_ − *p*_i_ represents the difference between hydrostatic pressures between microvasculature and interstitial space, and *π*_v_ − *π*_i_ is the difference in osmotic pressure across the wall. The first part of Equation 2 accounts for diffusion‐mediated macromolecular extravasation, and the second part accounts for bulk transport mediated extravasation. *P*, *L*, and *σ* are critical transport parameters that govern the tendency of a substance to undergo transvascular extravasation. Diffusion‐dependent extravasation depends upon the permeability of the wall to the solute, which is a function of both vascular characteristics and the xenobiotic of interest, in particular the size and surface charge of the solute relative to the capillary wall porosity and charge. Conductivity, a measure of how effectively fluid can extravasate into the interstitium, is a function of the porosity of the microvasculature, and determines the bulk‐transport mediated extravasation of macromolecules based on the reflection coefficient of the wall, which is a function of the relative size and surface charge of the solute and capillary wall pores. (As a reference, while free drugs are generally <1 nm in size, mAbs are generally ≤10 nm wide, and NPs may range in diameter from 1–100 nm; the size of microvascular fenestrations may vary from 1–5 nm in brain, lungs, and muscle, ~6–10 nm in kidneys, ~180–280 nm in liver, and ~5 µm in spleen (Sarin, 2010)). Of note, the build‐up of interstitial fluid pressure due to poor lymphatic drainage and solid stress due to growth in a tumor causes the hydrostatic pressure difference between the vasculature and interstitium to diminish. As a result, diffusion is the key mechanism of macromolecular extravasation in tumors (R. K. Jain & Stylianopoulos, 2010). Further, as demonstrated by Wittrup et al., the concentration of macromolecules attained in the tumor interstitium is much lesser than the plasma (systemic) concentration of macromolecules due to permeability being the limiting factor in the diffusive extravasation of macromolecules (Thurber et al., 2008).

Once macromolecules have crossed the capillary wall into the interstitium, they are transported via bulk transport with the interstitial fluid, or through diffusion to reach the target cells, represented as (R. K. Jain & Stylianopoulos, 2010):

(3) $$\frac{\partial C_{i}}{\partial t}+v\nabla C_{i}=D\nabla^{2}C_{i}+R$$

where *v* is the interstitial fluid velocity, *D* is the diffusion coefficient of the macromolecules, and the term *R* accounts for the sinks that cause macromolecular binding and degradation in the interstitium. However, due to elevated interstitial fluid pressure and solid stress, diffusion is the primary mechanism of transport in the tumor interstitium (Boucher & Jain, 1992; Pascal et al., 2013; Stylianopoulos et al., 2013). As a result, diffusion is the primary mechanism of transport in the tumor interstitium (Pascal, Bearer, et al., 2013). Finally, once in the proximity of cells, macromolecules maybe internalized through clathrin‐dependent/independent endocytosis,caveolin‐dependent/independent endocytosis, or receptor‐mediated endocytosis, depending upon their size and surface characteristics (Belleudi et al., 2012; Perera et al., 2007; S. Zhang, Gao, & Bao, 2015; Xiao, & Gan, 2013).

As described, many factors during the transvascular and interstitial transport ultimately affect the accumulation of macromolecules in tumors, demonstrating why plasma concentration kinetics cannot serve as an accurate surrogate for the tumor site concentration kinetics, and highlighting the importance of estimating macromolecular concentration at the site of action. In vivo imaging is a powerful tool to visualize the dynamics of macromolecules and to understand their transport via the processes involved in delivery to the site of action, which may help to overcome the limitations of plasma concentration kinetics in PK studies. Imaging can also help quantify the various parameters involved in the transport process, and thus guide the development of mathematical models for descriptive and predictive purposes. We now discuss state‐of‐the‐art imaging modalities that are utilized to investigate the pharmacology of macromolecules.

## 3 STATE‐OF‐THE‐ART IMAGING MODALITIES FOR NPs AND mABs

### 3.1 Magnetic imaging

**FIGURE 3:**
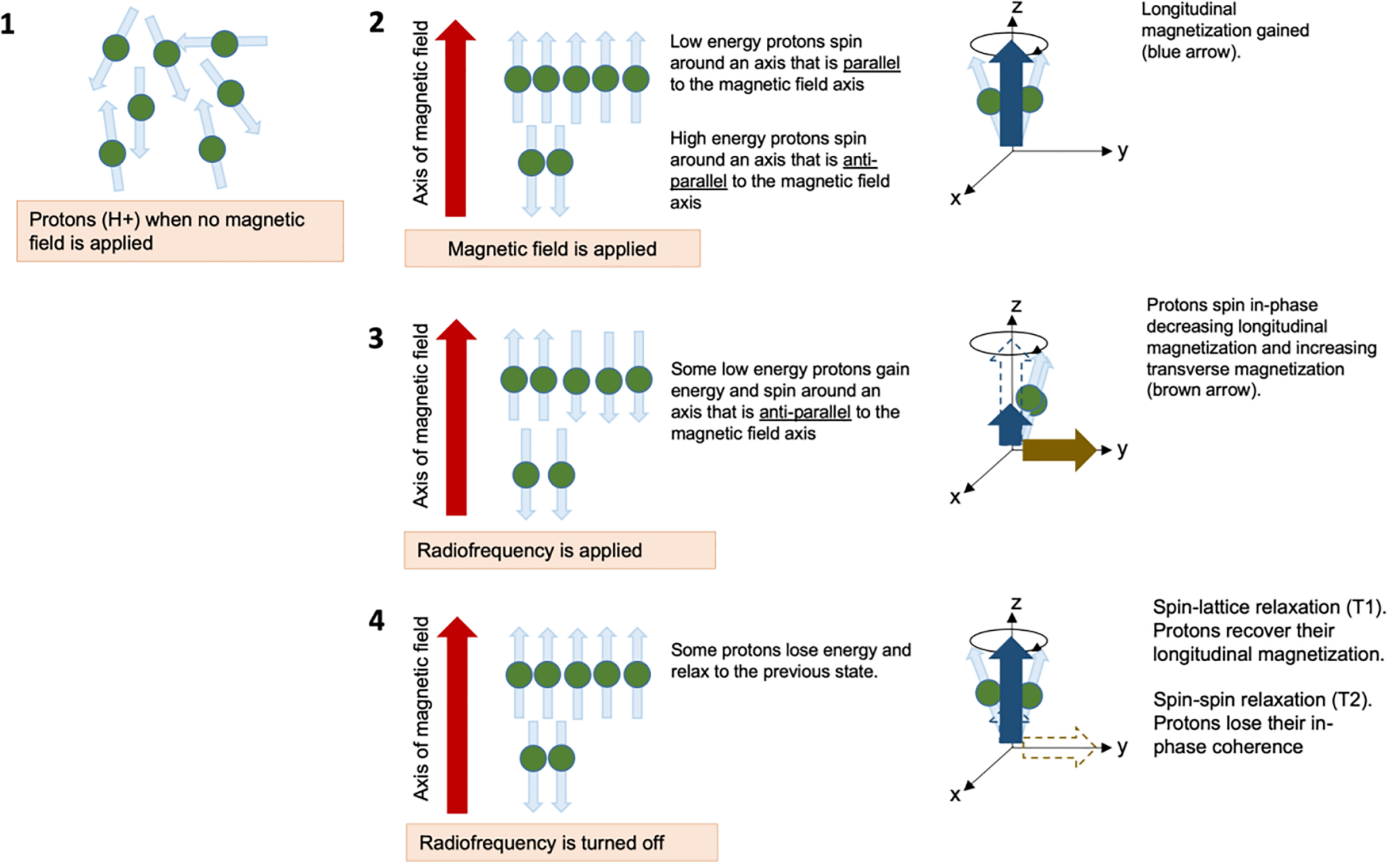
Schematic representation of nuclear magnetic resonance, the working principle of magnetic resonance imaging

Over the past decades, the application of magnetic resonance imaging (MRI) has expanded significantly in clinical practice. MRI is a non‐ionizing imaging modality that uses a strong magnetic field and radio waves to produce detailed images of anatomical structures. It is based on the phenomenon of nuclear magnetic resonance (NMR), which involves the perturbation of spin‐alignment of nuclei in a strong magnetic field through the application of radio frequency (see Figure 3). The nuclear spin of atoms in a strong and constant magnetic field is either oriented parallel or anti‐parallel to the external magnetic field. When this system is subject to an electromagnetic pulse (e.g., radio waves) at resonance frequency (also called Larmor frequency), it excites the nuclei to a higher energy state such that their spins are flipped and oriented anti‐parallel to the magnetic field, and are now referred to as being in resonance with the magnetic field, hence the name NMR. Subsequently, when the electromagnetic field is switched off and the nuclei relax, they revert to their equilibrium state (parallel spin orientation) that is accompanied by the release of energy in the form of radio waves that can be captured by antennas to produce an image (Bushberg & Boone, 2011). Due to the abundance of water and fat in biological tissues, clinical MRI is based on the NMR of hydrogen ions (protons). MRI produces high‐resolution images by measuring the spin magnetization of protons and their respective longitudinal (T1) and transverse (T2) relaxation rates. MRI provides high spatial resolution and sufficient penetration depth for clinical imaging, with high contrast‐to‐noise ratio but low sensitivity (Figure 1). Paramagnetic contrast agents (refer to Table 1) that interfere with local magnetism are often injected into the patient, resulting in changes in proton density and spin characteristics that enhance the contrast of MRI images.

**TABLE 1:** Summary of key magnetic resonance imaging (MRI)‐contrast enhancement agents and the corresponding macromolecules investigated through MRI

Commercial names of contrast agents with *t*_1/2_	NPs and mAbs investigated preclinically or clinically	References
Gadolinium‐based probes		
MultiHance (*t*_1/2_ = 1.6 hr) Gadovist (*t*_1/2_ = 1.7 hr) Eovist (US)/primovist (EU) (*t*_1/2_ = 0.9 hr) Dotarem (*t*_1/2_ = 1.6 hr)	NPs: Liposomes, silica, gold, Gd_2_O_3_‐cyclodextrin‐folic acid, upconversion NaGdF_4_–CaCO_3_ mAbs: Anti‐NG2, ‐CD33, ‐MUC1	Detappe et al. (2017), Mortezazadeh et al. (2019), Sharma et al. (2012), and Warram et al. (2014)
Iron oxide‐based probes		
Feridex (*t*_1/2_ = 2.4 hr) Resovist (*t*_1/2_ = 4.5 min and 3 hr) Feraheme (*t*_1/2_ = 15 hr)	NPs: Dextran@Fe_3_O_4_, carboxydextran@Fe_3_O_4_, carboxydextran@ultrasmall Fe_3_O_4_ (Ferumoxytol), MnO–Fe_3_O_4_; mAb: Anti‐HER2	Hope et al. (2015), Warram et al. (2014), and Xu et al. (2019)

Abbreviations: mAb, monoclonal antibody; NP, nanoparticle; *t*_1/2_, circulation half‐life.

MRI has been applied both preclinically and clinically for non‐invasive pharmacological assessment of macromolecules intended for applications in cancer treatment. Alsaid et al. (2017) used contrast enhanced MRI to study the impact of a mAb AccretaMab (anti HER3) on its HER3 positive tumor growth inhibitory effects in human xenograft tumor‐bearing mice. Using ultrasmall superparamagnetic iron oxide (USPIO) NPs (ferumoxytol) as contrast agent, they were able to detect immune system anti‐tumor response in the form of macrophage recruitment in mice treated with the novel antibody in contrast to the negative control, as assessed by greater reduction in signal‐to‐noise ratio after ferumoxytol injection. Palm et al. (2003) studied the in vivo disposition kinetics of trastuzumab mAb (anti‐HER2/neu) in mice bearing ovarian carcinoma using positron emission tomography (PET) imaging coregistered with standard MRI. Co‐registration with MRI was done to confirm the site of trastuzumab uptake (intra‐tumoral). The study revealed rapid systemic uptake of the mAbs following intraperitoneal injection, followed by tumor localization. Ramanathan et al. (2017) used the tumor deposition characteristics of ferumoxytol, evaluated through ferumoxytol enhanced MRI, to assess the therapeutic efficacy of a nanoliposomal irinotecan formulation in patients with advanced solid tumors. Their findings suggest a significant positive correlation between tumor accumulation of ferumoxytol at early time points and tumor regression caused by the therapeutic NPs. Together, these studies demonstrate the applicability of ferumoxytol enhanced MRI as a noninvasive biomarker to predict the efficacy of NP‐based drug delivery in solid tumors.

The challenges of using MRI in assessing the therapeutic effect of macromolecules include long image acquisition time and low sensitivity. Contrast‐enhanced MRI is also limited by rapid clearance of the contrast agent, and images obtained through this modality usually can only be collected for a short time window. Additionally, in the case of the work presented here, super paramagnetic iron‐oxide (SPIO) NP‐based MRI gives dark signals in the ROI that are sometimes difficult to differentiate from artifacts or local field inhomogeneity.

### 3.2 Nuclear imaging

Nuclear imaging modalities such as PET and single‐photon emission computed tomography (SPECT) utilize the γ‐ray signal emitted from radionuclides to visualize macromolecules in vivo. The superior properties of the γ‐ray signal, including excellent sensitivity, no limitation on tissue penetration, and facile quantification, have made nuclear imaging a prominent non‐invasive imaging tool (Figure 1). With established radiolabeling technologies and the increased availability of radionuclides approved by the U.S. Food and Drug Administration (FDA) (Table 2), great interest has been spurred to deploy nuclear imaging for pre‐clinical assessment of the PK of macromolecules (F. Chen et al., 2016; Goel et al., 2019; Madru et al., 2012; Niemeijer et al., 2018).

**TABLE 2:** Summary of key radioisotopes and the corresponding macromolecules investigated through nuclear imaging

Commercial names of probes with *t*_1/2_ a and *D*_1/2_ a	NPs and mAbs investigated preclinically or clinically	References
Radioisotopes for PET imaging		
18F		
Neuraceq (*t*_1/2_ = 1 h, *D*_1/2_ = 1.82 h)	NPs: PLGA, porous silicon, gold mAb: anti‐PD‐L1	Devaraj, Keliher, Thurber, Nahrendorf, and Weissleder (2009), Keinanen et al. (2017), Sirianni et al. (2014), and Zhu et al. (2014)
64Cu		
^64^Cu‐CHX (*D*_1/2_ = 12.7 h)	NPs: PS‐PEG, liposomes/liposomes‐nucleic acid, Mn_3_O_4_‐PEI, QDs, MWCNT, nanographene mAb: anti‐PD‐L1, ‐PD‐1, ‐CTLA‐4, ‐mesothelin, ‐CD105 (aka TRC105)	Du et al. (2019), Guo et al. (2015), Kobayashi et al. (2015), D. E. Lee, Choi, et al. (2013), Z. Liu et al. (2007), Mukai et al. (2019), Shi et al. (2017), Zhu et al. (2018)
89Zr		
89Zr‐CHX (*D*_1/2_ = 3.27d)	NPs: Cerium oxide, MWCNT, liposomes mAb: Trastuzumab	Abou et al. (2013), Heskamp et al. (2017), McDonagh et al. (2018), Ruggiero et al. (2010)
Radioisotopes for SPECT imaging		
99mTc		
Sestamibi (*t*_1/2_ = 48 h, *D*_1/2_ = 6 h)	NPs: Folate‐PGA with chitosan or PEG, PAMAM dendrimers, silica, gold, Co4L6 nanocage mAb: Anti PD‐L1	Dumoga et al. (2017), Morales‐Avila et al. (2011), Rainone et al. (2017), Song et al. (2017), Xing et al. (2019)
111In		
ProstaScint (*D*_1/2_ = 2.81d)	NPs: Mesoporous silica, liposomes, micelleplex PEI‐PLC‐PEG‐FA mAb: Anti‐PD‐L1, ‐PSA (prostate specific antigen)	Banerjee et al. (2017), Chatterjee, Lesniak, and Nimmagadda (2017), Dogra et al. (2018), Helbok et al. (2010), Jones, Douglas, Shields, and Merkel (2018)
131I		
^131^I‐CHX; *t*_1/2_ = 35 h	NPs: Polymerosome, PLA–PEG, silver mAb: Tositumomab (Bexxar®)	J. Cao et al. (2019), Chrastina and Schnitzer (2010), Kaminski et al. (1996), Press et al. (1993)

Abbreviations: PEI, polyethyneimine; PS, polystyrene; PLA, poly(lactic acid); PLGA, poly(lactic‐co‐glycolic acid); PEG, polyethylene glycol; PGA, polyglutamic acid; MWCNT, multi walled carbon nanotubes; QDs, quantum dots; PGA, pteroylglutamic acid; *t*_1/2_, circulation half‐life; *D*_1/2_, radioactive decay half‐life.

a If available.

Specific radionuclides, such as technetium‐99m (Madru et al., 2012), copper‐64 (^64^Cu) (Davis et al., 2017), indium‐111 (Dogra et al., 2018; Josefsson et al., 2016; Lindenberg et al., 2015), and zirconium‐89 (^89^Zr) (Börjesson et al., 2009; Dijkers et al., 2010; Pérez‐Medina et al., 2015), are preferred to conduct PK studies of macromolecules (Farzin, Sheibani, Moassesi, & Shamsipur, 2019) because of similarities between the timescale of the PK processes and the decay half‐life of the radionuclides. For instance, using ^89^Zr‐labeled PET imaging, specificity of high‐density lipoprotein NPs for tumor‐associated macrophages and their PK was assessed in an orthotopic mouse model of breast cancer (Pérez‐Medina et al., 2015). Meanwhile, F. Chen et al. (2013) pioneered the use of ^64^Cu‐labeled silica NPs to image tumor‐specific delivery with PET. In addition, it became feasible to establish the effects of NP properties on their in vivo disposition using imaging‐based PK derived from in vivo SPECT/CT imaging and ex vivo radiation dosimetry biodistribution (F. Chen et al., 2013; Dogra et al., 2018). Similarly, PET and SPECT have also been largely applied to determine the optimal dosage, biodistribution, and tumor uptake of mAbs in cancer patients. Of note, quantification of uptake in patients with metastatic breast cancer was feasibly assessed for ^89^Zr‐labeled mAb trastuzumab using PET imaging (Dijkers et al., 2010), ^89^Zr immuno‐PET imaging was practiced to assess safety of ^89^Zr‐labeled mAb U36 in patients with head and neck squamous cell carcinoma (Börjesson et al., 2009), and PK studies of PD‐1/PD‐L1 mAb using radiolabeled PET/SPECT highlighted new opportunities to optimize and monitor the efficacy of immune checkpoint blockade therapy (Josefsson et al., 2016; Maute et al., 2015; Natarajan et al., 2015; Teng, Meng, Kong, & Yu, 2018).

Obtaining highly precise measurements of macromolecule distributions via PET/SPECT imaging may be compromised by in vivo integrity or stability of the radiolabeling, which can result in dissociation of the radiolabel molecules from the macromolecules of interest, as these dissociated radioisotope molecules are still detected by the ultrasensitive γ‐ray signal (Goel, Chen, Ehlerding, & Cai, 2014). The mismatched biodistribution between macromolecules and the dissociated radionuclides may lead to incorrect assessment of macromolecule biodistribution from the in vivo imaging data. Also, high uptake of free ^64^Cu in liver and bladder, and significant uptake of free ^89^Zr in bones when dissociated from unstable radiolabeled macromolecules has been reported and can pose challenges in accurate assessment of PK (Deri, Zeglis, Francesconi, & Lewis, 2013; Y. Wang et al., 2012; Y. Zhang, Hong, & Cai, 2011). To overcome this problem, efforts were devoted to develop chelate‐free radiolabeling technologies, such as intrinsically radiolabeled NPs (F. Chen et al., 2015; Wall et al., 2017), protected surface coatings for radio‐stability of NPs (Frellsen et al., 2016). Also, novel chelators were developed for stable radionuclide‐mAb conjugates (Deri et al., 2014; Zhai et al., 2015). In any case, radiolabeling must be approached carefully, as radiolabeling may modify the surface properties of macromolecules and thus affect their PK. For example, systematic study of ^64^Cu‐labeled engineered antibody fragment ch12.18‐ΔC_H_2 with various chelators showed increased uptake in kidney with increased net positive charge of radiolabeled chelators (Dearling et al., 2015). Also, it is worth noting that, in practice, the fundamental spatial resolution limits of nuclear imaging often necessitate coscanning with an anatomical imaging modality such as CT. This spatial resolution limit may also be overcome by using integrated PET with MR multimodality imaging, which enables coregistration of morphologic and multifunctional information (Schlemmer et al., 2008). In yet another approach, Wall et al. (2017) attempted to expand the macroscopic resolution at organ level by integrating PET imaging with surface‐enhanced resonance Raman scattering imaging to obtain high‐precision intraoperative imaging.

### 3.3 Optical imaging

**FIGURE 4:**
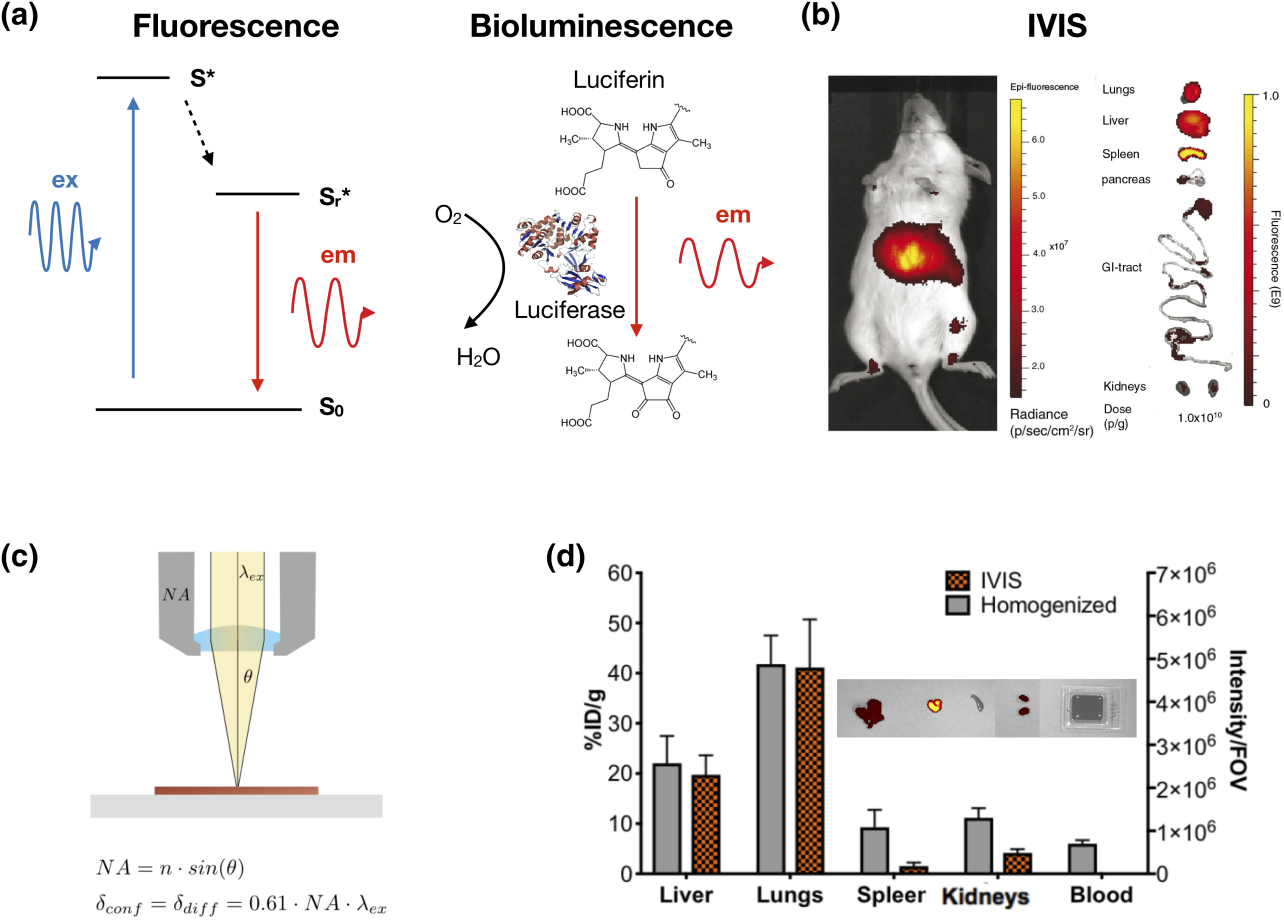
Non‐invasive optical imaging methods to evaluate in vivo biodistribution. (a) Principles of fluorescence and bioluminescence imaging. (b) Representative in vivo and ex vivo fluorescence imaging (IVIS Spectrum). (c) Geometrical demonstration of Abbe's diffraction limit for optical methods. (d) Biodistribution of fluorescent porous silica microdisks estimated via fluorescent signal (IVIS spectrum) vs quantitative optical imaging (homogenized organs). Left *y*‐axis refers to %ID/g as measured with quantitative optical imaging, right *y*‐axis to fluorescence intensity as measured via IVIS. Results are shown after background subtraction. (Reproduced with permission from Nizzero et al., 2019. Copyright 2019 Elsevier)

While their clinical application remains challenging, optical in vivo imaging systems (IVOI) are widely employed in preclinical studies to evaluate the biodistribution of a variety of compounds, including mAbs, cells, NPs, and microparticles (Martelli, Lo Dico, Diceglie, Lucignani, & Ottobrini, 2016). This is accomplished by fluorescent or bioluminescent labeling onto the entities that are to be visualized or tracked, and is often advantageous over other labeling approaches previously discussed due to the ease of conjugation and broad availability of commercial compounds and modification protocols to label non‐fluorescent macromolecules. The two main imaging modalities used in IVOI rely on the corresponding physical phenomena of fluorescence and bioluminescence. The working principle of Stoke's shift fluorescence emission is reported in Figure 4a (for brevity, we have chosen not to provide a detailed discussion of the mechanistic underpinnings of resonance fluorescence here), where a photon of energy *E* ~ *λ*_ex_^−1^ excites the ground state of a fluorescence compound (*S*_0_) to its excited state S*. Following non‐radiative relaxation to the radiative excited state *S*_r_*, a photon of energy *E*_em_ ~ *λ*_em_^−1^ is emitted. Similarly, the working principle of bioluminescence is reported in Figure 4b. In bioluminescence, the excitation energy is typically produced by a chemical reaction occurring between an enzyme (luciferase) and a light‐emitting molecule (called a substrate; e.g., luciferin) in the presence of oxygen, which is necessary for the enzyme to oxidize the substrate. Regardless of the excitation or emission mechanism, in IVOI applications, emitted photons are then captured by a detector that converts their energy into an electric signal proportional to the number of emitting molecules found in the sample.

IVOI techniques are well‐established methods to study how the tumoritropic or organotropic potential of macromolecules influences the concentration kinetics at the target site. Under certain conditions, 3D image processing tools can also be used to reconstruct the geometrical volumetric features of distribution of compounds or cells from IVOI data, as shown by Abdelwahab, Sankar, Preul, and Scheck (2011) for mouse glioma development. While some materials exhibit intrinsic luminescence or fluorescence, the majority of compounds of interest for medical applications require additional chemical modification or conjugation with optically active compounds (Table 3). Analogous to the radiolabeling molecules previously discussed, fluorescent source molecules must be carefully selected, and care must be taken to attach them to their labeled substrates without changing the substrate functionality. It is established that fluorescent labeling may potentially alter the in vivo compound biodistribution, as demonstrated by Cilliers, Nessler, Christodolu, and Thurber (2017) for two clinically approved antibodies, trastuzumab and bevacizumab. In another notable example, (Peterson, Wilson, Huang, Dimasi, and Sachsenmeier (2016) showed that in vivo antibody biodistribution results varied according to the near‐infrared (NIR) labeling fluorophore emission wavelength used.

**TABLE 3:** Summary of fluorescent probes and the corresponding macromolecules investigated through optical imaging

Fluorescent probes	NPs and mAbs investigated preclinically or clinically	References
Cyanine; Alexafluor; DyLight; AndyFluor; rhodamine; fluorescein; Licor IRDye	NPs: Liposomes, mesoporous silica/cornell dots, PLGA mAbs: anti PD‐L1 (Atezolizumab)	Kumar et al. (2010), J. J. Lee, White, Rice, and Smith (2013), Meng et al. (2018), Popovic et al. (2010), W. Zhu et al. (2019)

Abbreviations: PLGA, poly(lactic‐co‐glycolic acid); mAb, monoclonal antibody; NP, nanoparticle.

Despite wide use in preclinical studies, non‐invasive optical imaging methods suffer from the unavoidable attenuation effects due to interaction of emitted light with biological matter (Nizzero et al., 2019). While optical imaging resolution can be pushed down to the Abbe diffraction limit (Figure 4c) for confocal microscopy setups, the resolution for in vivo IVOI is generally poorer (Figure 1) (Arms et al., 2018). Approaches to minimize this attenuation effect include the use of NIR dyes (*λ*_ex_/*λ*_em_ = 600 nm–1,000 nm) that can penetrate biological matter up to a few cm of tissue (Coll, 2011; Y. Liu, Tseng, & Huang, 2012; Vats et al., 2017). Alternatively, quantitative results can be obtained with ex vivo tissue optical methods that measure fluorescence from homogenized organ tissues (Bernhard et al., 2018; Mattu et al., 2018; Oliveira et al., 2012; Wolfram et al., 2017).

However, these invasive methods are challenging to translate to clinical practice. This has been recently demonstrated in a new bioluminescent reporter compound that was developed for in vivo imaging of extracellular vesicles (Gangadaran et al., 2017). This ex vivo study revealed similar accumulation between lung and liver tissues, followed by spleen and kidneys, in contrast to data collected at similar time points via in vivo measurements that showed the highest accumulation occurs in lungs, followed by liver, kidneys, and then spleen. In a different study, Nizzero et al. (2019) also observed tissue attenuation effects in IVOI by directly comparing biodistribution of the fluorescent porous silica microdisks as estimated with IVIS Spectrum with the percent of injected dose (%ID) calculated by fluorescence measured in homogenized organs (Figure 4d). Notably, the two techniques differ in their ability to remove bias due to tissue attenuation of fluorescent signal. In fact, conversion to absolute units (%ID/g) is only possible with the homogenized organ technique, by calculating attenuation factors in each organ through the use of a calibration curve. This is not possible with IVOI, so relative units (such as radiance) must be used for comparison. Thus, while a helpful tool to quickly compare in vivo targeting capabilities and PK of macromolecules and different compounds (Arms et al., 2018), the validity of noninvasive in vivo optical imaging methods to evaluate biodistribution may likely be considered semi‐quantitative at best (Nizzero et al., 2019).

## 4 IMAGE‐GUIDED MATHEMATICAL MODELING

Mathematical modeling has been increasingly perceived as a valuable tool in advancing a variety of research fields in nanotechnology and nanomedicine (Dogra, Butner, et al., 2019). Models informed by proper parameter quantification and validated through testing with different forms of multidimensional data can be used to provide quantitative *mechanistic* insights into, and to establish important quantitative correlations between, the biological, biophysical, and biomechanical processes of a particular biological or clinical problem that occur on or across multiple scales in time and space. In the field of cancer research in particular, biologists and medical scientists have been using mathematical models to generate new experimentally testable hypotheses, predict complex behaviors of the biological system of interest, and optimize drug delivery and therapeutic effect, thereby providing potentially clinically useful insights into different treatment systems (Cristini, Koay, & Wang, 2017; Dogra et al., 2019; Z. Wang, Butner, Kerketta, Cristini, & Deisboeck, 2015; Z. Wang & Deisboeck, 2019).

Mathematical models of NP and mAb delivery and their therapeutic effects in tumors have been integrated with and informed by in vivo and in vitro imaging of biodistribution and tumor kill, and are yielding valuable insights into the mechanisms of action of these cancer therapies (Clancy et al., 2016; Haddish‐Berhane, Rickus, & Haghighi, 2007). As discussed previously, the delivery of macromolecules to the site of action can be discretized into three key steps: *i*) blood‐flow driven vascular transport to the organ of interest, *ii*) transvascular migration into the interstitium, and *iii*) and interstitial diffusion. The key parameters that characterize these processes can be quantified through in vivo imaging to parameterize mathematical models for quantitative pharmacological evaluation of macromolecules (see Table 4).

**TABLE 4:** List of key physiological parameters quantifiable through in vivo imaging

Parameter	Units	Imaging techniques	Source
Blood flow rate	ml/min	Doppler tomography; Tracer kinetics analysis of dynamic contrast enhanced MRI; functional MRI (specifically for cerebral blood flow)	Borogovac and Asllani (2012), Z. Chen et al. (1997), Henderson et al. (2000), O'Connor et al. (2011)
Vascular volume or blood volume fraction	ml	Dynamic contrast enhanced MRI	Henderson et al. (2000) and Hindel et al. (2017)
Extravascular volume	ml	Contrast enhanced MRI, computed tomography	Bandula et al. (2013), Kim, Savellano, Savellano, Weissleder, and Bogdanov (2004), Scully, Bastarrika, Moon, and Treibel (2018)
Permeability	cm/s	Fluorescence microscopy; dynamic contrast enhanced MRI	Henderson et al. (2000), Reyes‐Aldasoro et al. (2008)
Permeability‐surface area product	ml/min/100 ml	MRI; computed tomography; dynamic contrast enhanced MRI	Cha et al. (2006), R. Jain et al. (2008), Tofts et al. (1999), Weidman et al. (2016)
Lymph flow rate	ml/min	Doppler optical coherence tomography; ultrasound; MRI; computed tomography	Blatter et al. (2016), F. Zhang, Niu, Lu, and Chen (2011), J. Zhu et al. (2019), Ruiz‐Ramírez, Ziemys, Dogra, and Ferrari (2020)
Cellular uptake rate	1/s	Optical imaging	Ahmed et al. (2017), Au et al. (2009), Liao‐Chan et al. (2015), van der Zwaag et al. (2016)

Abbreviation: MRI, magnetic resonance imaging.

Derivation of prognostically correct and clinically meaningful information from imaging data often presents a significant challenge, in part due to the multiple interpretations that can arise from a single data set and lack of standardized data interpretation paradigms in many cases (Livingstone & Salt, 2005). This may be overcome by the construction of semi‐mechanistic or mechanistic mathematical models that not only fit the observations, but that can also offer some degree of predictive power (Brooks & Tobias, 1996). To date, a variety of mathematical models have been applied to the study of NP and mAb biodistribution kinetics, including physiologically based pharmacokinetic (PBPK) models (Abuqayyas & Balthasar, 2012; Dogra et al., 2018; Kuepfer et al., 2016; Li, Al‐Jamal, Kostarelos, & Reineke, 2010; Pascal, Ashley, et al., 2013; Shah & Betts, 2012; Z. Wang, Butner, Cristini, & Deisboeck, 2015) and other novel mechanistic models (Haddish‐Berhane et al., 2007; Sciumè et al., 2012; Sciumè et al., 2013), which are engineered to possess particular advantages over the others, depending on the nature of the problem under investigation.

A PBPK model is a mechanistic model that consists of a network of compartments that represent physiological volumes of the biological ROI, such as an organ, a tissue, or a tumor. These systems exchange quantifiable properties of interest, for example, blood, nutrients, drug molecules, macromolecules, etc., with a specific set of neighboring compartments, as determined by the underlying anatomy. Possible mechanisms of transport between the connected compartments include diffusion (Godin et al., 2010), convection (Y. Cao, Balthasar, & Jusko, 2013), or their combination, where the spatial dependency is homogenized through a lumping process that consists of integration over space, effectively reducing bivariate components (space and time) to time‐dependent averages (Thompson & Beard, 2011). Once the PBPK network has been defined, the complex reactions that take place within each compartment, and their interactions with and effects on surrounding neighbors, are described with a set of time dependent equations. PBPK models are easy to implement and modify (McNally, Cotton, & Loizou, 2011), robust numerical tools exist to solve them (Blancato, 1994), and several simulation and development packages that facilitate their implementation, sharing, and testing in the scientific community are available (Lin et al., 2017). Consequently, this has led to the wide acceptance of PBPK methods in the pharmaceutical industry (Rowland, 2013). However, this simplicity comes with a price, the most relevant is that standard PBPK methods are not suitable to model problems containing more than one independent variable, for example, systems that have a strong dependency on and significant variation in time, space, and potentially additional material dimensions. Nonetheless, efforts have been made to partially extend their range of applicability, inducing methods that use hybrid models that combine computational fluid dynamics (CFD) and PBPK modeling techniques (Andersen et al., 2000; Corley et al., 2009). To date, various imaging techniques have been successfully incorporated into the conception of these models, including SPECT, PET, fluorescence imaging, and MRI (Dogra, Butner, et al., 2019; Ng et al., 2020).

**FIGURE 5:**
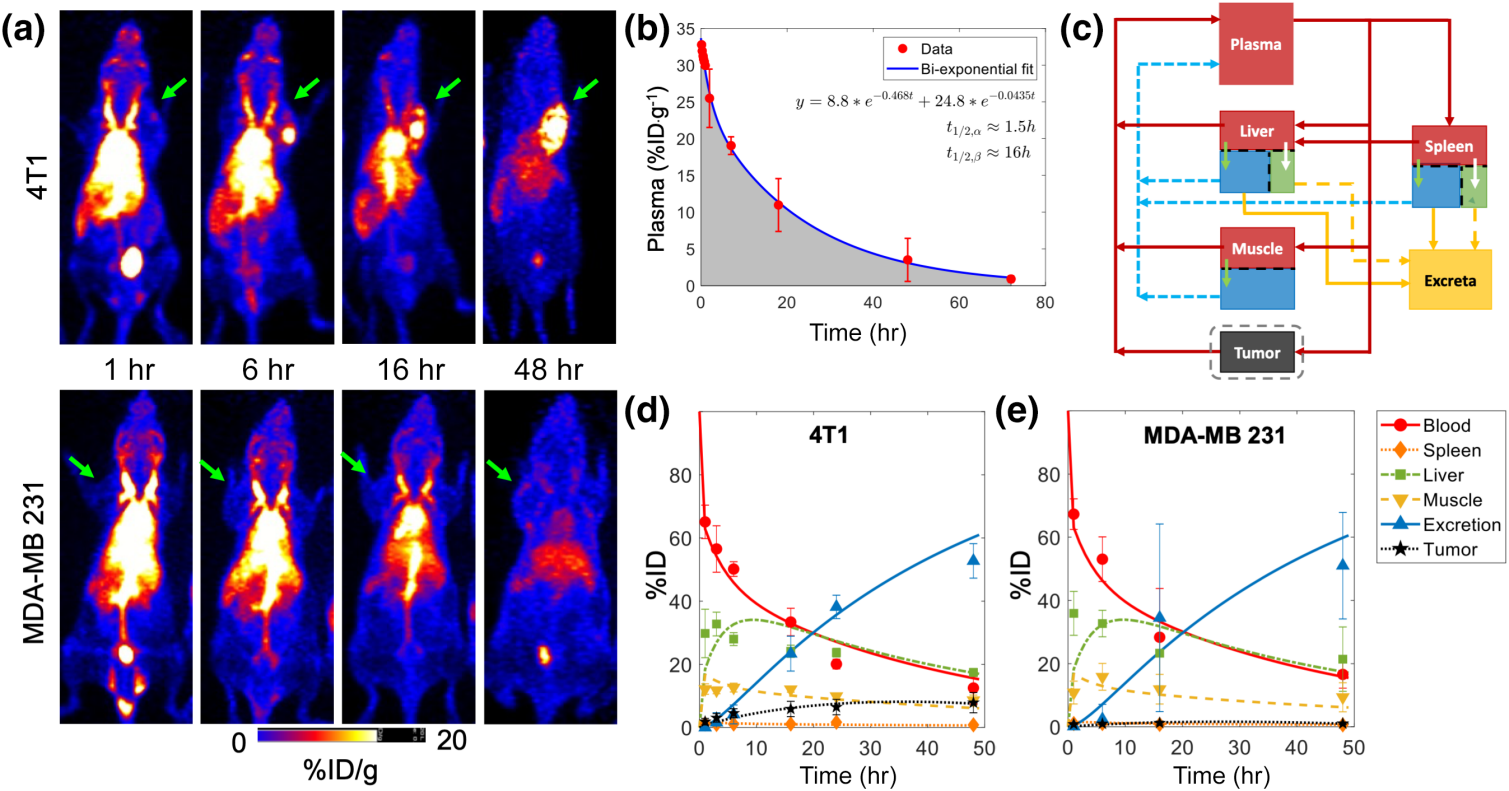
Noninvasive imaging‐guided mathematical modeling to evaluate the pharmacokinetic (PK) of ultrasmall porous silica nanoparticles (UPSNs). (a) Representative longitudinal positron emission tomography (PET)/computed tomography (CT) images of 4T1 (upper panel) and MDA‐MB 231 (lower panel) tumor‐bearing mice injected with ^64^Cu‐labeled ultrasmall porous silica nanoparticles (UPSNs). (b) Two‐compartment PK modeling of plasma concentration kinetics data of UPSNs obtained in healthy mice. (c) Schematic of reduced physiologically based pharmacokinetic (PBPK) model to investigate the disposition kinetic of UPSNs in tumor‐bearing mice. (d, e) PBPK‐model fits obtained through nonlinear regression of the data. (Reproduced with permission from Goel et al., 2019. Copyright 2019 Wiley)

We recently demonstrated the application of image‐guided mathematical modeling to investigate the in vivo disposition of ultrasmall porous silica NPs in two mouse models of breast cancer (Figure 5) (Goel et al., 2019). This was accomplished with a reduced PBPK model comprised of systemic circulation, liver, spleen, muscle, and tumor compartments. Literature‐derived blood and lymph flow rates were used to model the whole‐body transport of NPs, and quantified PET/CT imaging data was used to estimate empirical transport parameters related to the tumor compartment and phagocytic uptake rates of NPs in the liver and spleen. The study revealed that despite similar systemic circulation behaviors, the NPs demonstrated different tumor uptake profiles, which correlated with the degree of vascularization of the tumors, as confirmed through histopathological analysis (Goel et al., 2019).

**FIGURE 6:**
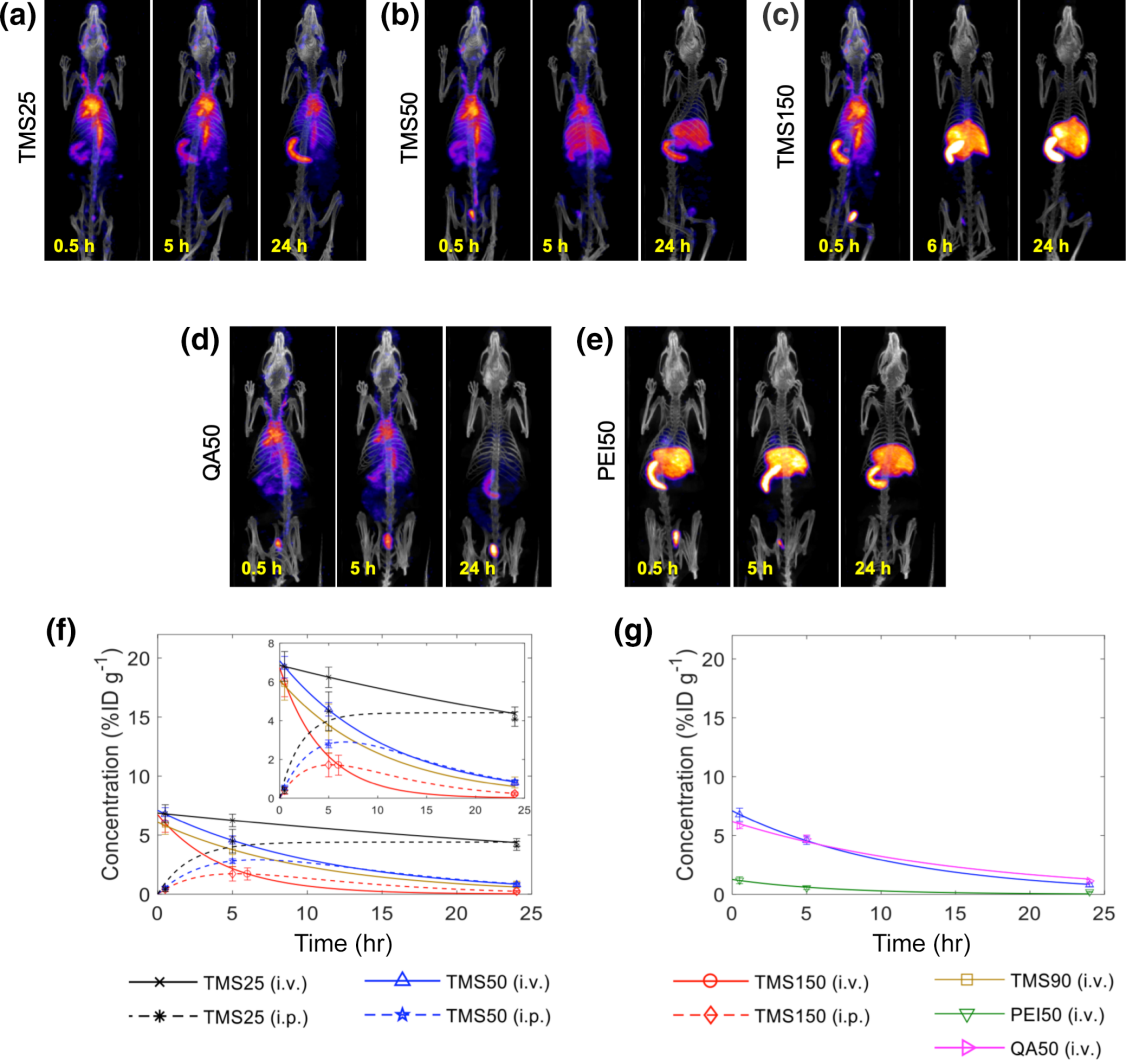
Representative single photon emission computed tomography/CT (SPECT/CT) images showing the whole‐body spatiotemporal evolution of trimethylsilane (TMS)‐, quaternary amine (QA)‐, and polyethylene imine (PEI)‐coated mesoporous silica NPs of variable sizes and surface charges (a) TMS25: 25 nm, −5 mV, (b) TMS50: 50 nm, −7 mV, (c) TMS150: 150 nm, −4 mV, (d) QA50: 50 nm, +38 mV, and (e) PEI50: 50 nm, +37 mV, in vivo. (f, g) Model fits to the longitudinal systemic concentration kinetics data obtained from quantified SPECT/CT images of the heart region‐of‐interest. (Reproduced with permission from Dogra et al. (2018). Copyright 2018 Springer Nature)

Our group has also previously developed simplistic, semi‐mechanistic mathematical models based on quantified in vivo SPECT/CT imaging data to establish the relationship between physicochemical properties (size and surface chemistry) of mesoporous silica NPs (MSNs) and their in vivo pharmacokinetics (Figure 6) (Dogra et al., 2018). The study revealed a monotonic decline in systemic bioavailability of NPs with systematic increase in particle size from ~32 to ~142 nm, independent of the route of administration. This was accompanied by a simultaneous increase in NP accumulation in the liver and spleen. The study also highlighted the importance of surface chemistry in governing the biodistribution profile of MSNs, revealing that MSNs with surface exposed amines (polyethylene imine, PEI) had a much shorter circulation half‐life than size‐ and charge‐matched MSNs with shielded surface amines (quaternary amine, QA), due to rapid sequestration of the former in liver and spleen. Using the mathematical models, we were able to quantify important PK parameters and establish functional correlations between particle properties and biodistribution parameters (Dogra et al., 2018). Further, we also used the above data to develop a novel PBPK model to predict NP pharmacokinetics and tumor delivery in vivo. Through local and global parameter sensitivity analyses, we identified NP degradation rate, tumor blood viscosity, NP size, tumor vascular fraction, and tumor vascular porosity as the key parameters in governing NP kinetics in the tumor interstitium (Dogra et al., 2020).

Notable strides in the development of novel mechanistic mathematical models informed by imaging data have been achieved in recent years (T. A. Brocato et al., 2018, 2019; Cristini et al., 2017; Deisboeck, Wang, Macklin, & Cristini, 2011; Pascal, Bearer, et al., 2013; Z. Wang & Deisboeck, 2014; Zhihui Wang et al., 2016). To understand how NPs may be designed for optimal tumor capture and retention, T.‐R. Lee, Na, et al. (2013) examined how size affects bloodborne NP accumulation near the vasculature wall (a key factor in NP extravasation) with a mechanistic immersed finite element methods model, and compared model results against intravital video microscopy data in murine models. By representing NPs as Lagrangian solids in Eulerian fluid using a Navier–Stokes transport representation hybridized with discrete Mooney‐Rivilin material model representations of red blood cells (RBCs), their model provided valuable mechanistic understanding into why submicron sized NPs (500–1,000 μm) achieve increased incidence with the vasculature wall via displacement by RBC movement, in good agreement with intravital microscopy observations. Their results suggest NP designs that may increase the likelihood of delivery to tumors through vasculature fenestration‐mediated extravasation, albeit at a cost of using larger NPs than may be likely to penetrate and deliver therapeutic cargo deep into tissues.

**FIGURE 7:**
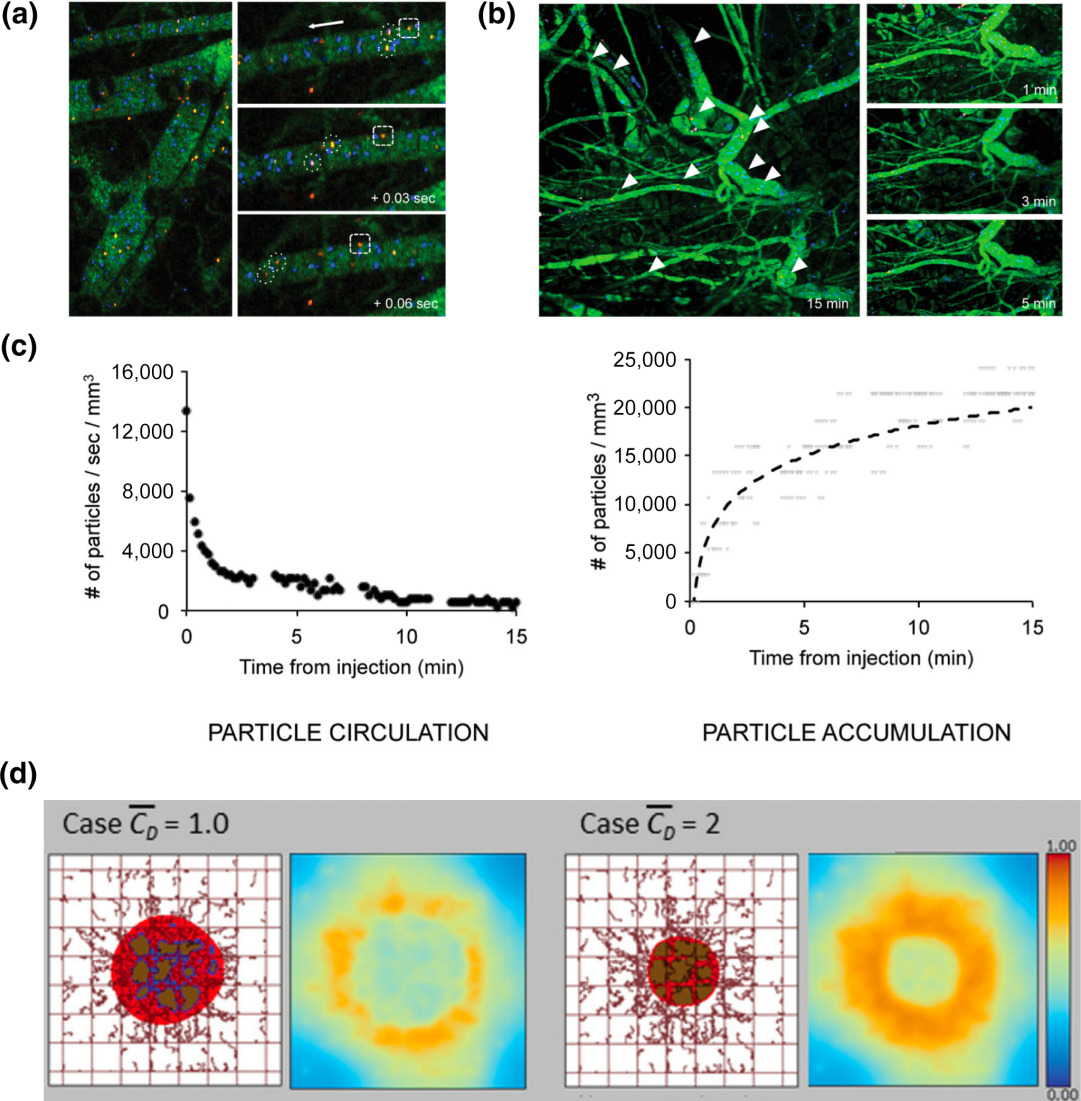
Intravital microscopic images of NP circulation in (a) healthy and (b) tumorous vessels. (c) Quantification of NP circulation and accumulation kinetics. (d) Computational modeling of therapeutic efficacy of NP‐delivered doxorubicin in a virtual tumor under different initial conditions. (Reproduced with permission from van de Ven et al. (2012). Copyright 2012 AIP Publishing)

After NPs are retained in tumor vasculature, they must achieve cargo delivery, often through the enhanced permeability and retention (EPR) mechanisms, whereby “leakiness” in tumor vasculature due to defects in tumor vascular epithelial cells and epithelial fenestrations, as well as the lack of lymph drainage from tumor vasculature (among other mechanisms), tends to increase macromolecular accumulation in tumors relative to other tissues. van de Ven et al. (2012) implemented a mechanistic model of tumor kill due to NP extravasation and subsequent drug delivery, including mathematical descriptions of NP extravasation (the EPR effect), cellular uptake, drug release (NP decay rate), and subsequent drug diffusion into the tumor. Model parameters were quantified using experimental values from intravital microscopy imaging of in vivo NP distribution in human B16 melanoma murine model cells and from literature‐derived values, yielding valuable quantification of actual drug delivered into cancer cells via NP dosing (Figure 7). Unfortunately, this result demonstrated that single administration of NPs was unable to deliver the IC_50_ for these tumor cells, suggesting that important further studies are needed to optimize dosing schedules and protocols for effective tumor kill. This model was later adapted and applied to an in vitro study of two‐ and three‐layer NP delivery of paclitaxel or cisplatin in human NSCLC monolayer/spheroid cultures by Curtis, England, Wu, Lowengrub, and Frieboes (2016), allowing the authors to quantitatively explain how longer half‐life and steady NP release rates of paclitaxel enabled increased tumor kill rates over NP delivered cisplatin; however it remains unclear how well these results may translate into in vivo models.

Additional modeling insights into the EPR effect on liposomal NPs were provided by Stapleton et al. (2013) who developed a mechanistic intra‐tumoral transport model (ITTM), built on the biophysical transport of pressure‐driven fluid flow through tissues and across the vasculature endothelium, which was validated against CT imaging data from two xenograft mouse models and one syngeneic rabbit tumor model. This model was shown to accurately predict imaging‐derived experimental data (*R* ^2^ > 0.9) for tumor NP accumulation due to EPR effects, however at the cost of neglecting the effects of variations in intratumoral interstitial fluid pressure, which is known to resist NP tumor penetration and lead to heterogeneous intratumoral NP delivery (Holback & Yeo, 2011). Hosoya et al. (2016) applied a mechanistic model of drug transport to theranostic doxorubicin‐containing heat sensitive‐based liposomes (HSL) embedded in targeted hydrogels, which were delivered in vivo into tumor‐bearing mice. Using imaging‐based tracking of NPs by replacing drug cargos with Gd‐DTPA and NIR dye to inform model parameters, these experiments revealed that HSL containing hydrogels showed a marked ~700% increase in tumor kill rates relative to traditional HSL delivery, confirming the predictions of their modeling work. While this model captures the pharmacodynamic effects of delivered drug, it only describes the effects of the NP delivered cargo and does not represent the NPs themselves, limiting its ability to quantitatively study many factors unique to NPs.

## 5 CONCLUSION

Advancements in in vivo imaging have enabled extensive pharmacological characterization of NPs and mAbs. Using state‐of‐the‐art imaging modalities, appropriately labeled macromolecules can be visualized over time throughout the body, which can allow the quantification of their disposition kinetics, facilitate the estimation of transport and physiological parameters, and help guide the development of mathematical models that can be employed for descriptive and predictive applications. Among the available macromolecule imaging modalities, we have chosen to focus only on those most commonly used in current practice, including MRI, SPECT, PET, and optical imaging. While MRI, PET, and SPECT are being used clinically, optical imaging has yet to be successfully transitioned to clinical use; nevertheless optical imaging is a widely used tool for preclinical development of macromolecules. Noninvasive whole‐body imaging not only provides a more comprehensive understanding of the disposition of macromolecules than traditional plasma concentration kinetics‐based pharmacological methods, but also sheds light on the underlying physiological changes in the diseased tissue, thereby opening avenues for the development of novel imaging‐based biomarkers for easy and early noninvasive disease diagnosis and improved prognosis. This also allows for the development and accurate parametrization of mathematical models based on correct biological and physical processes, in order to gain increased understanding of the key processes involved and increase understanding on how macromolecules may be designed to maximize their intended therapeutic effects.

Despite the remarkable potential of imaging‐based pharmacological assessment of macromolecules, there are caveats that must be considered during the quantification and interpretation of the imaging data obtained. It is essential to recognize what methodologies generate truly quantitative results (e.g. magnetic and nuclear imaging) that do not suffer from systematic errors (e.g. tissue attenuation for optical imaging). The stability of the bond between the imaging agent and the macromolecule it labels is critical for the accurate estimation of disposition kinetics and parameter values, and the breaking of this bond can result in leakage of the label from the macromolecule, confounding observations and providing inaccurate pharmacological assessments. The disposition behavior of macromolecules at early time points following administration is critical to accurately assess the pharmacokinetics of macromolecules, hence imaging modalities with low temporal resolution should be avoided in such circumstances, and are appropriate when only a semi‐quantitative assessment is required. Similarly, limitations of spatial resolution should be considered based on the nature of the assessment.

The integration of in vivo imaging with mathematical modeling is a valuable tool that, in addition to the pharmacological evaluation of macromolecules, can provide insights into mechanisms of transport barrier‐induced drug resistance or therapy failure, which can support the development of novel strategies to improve delivery and personalize treatment. Selection of which biological and physical phenomena should be included in such mathematical models, and their quantification, is improved when these are informed based on imaging‐based parameter estimates (rather than best fit estimates), which can support the development of mechanistic models for highly accurate predictive applications. Such models will not only provide greater insights into the complex interplay between pathophysiological states and the underlying transport mechanics, but will also serve as in‐silico tools to guide the design optimization of macromolecules for improved delivery and therapeutic efficacy. Further, the application of PBPK‐like modeling frameworks can provide reliable projections for human applications based on models developed for lower species (Aborig et al., 2019; Shah & Betts, 2012; Zhao, Cao, & Jusko, 2015). Taken together, these exciting advances are expected to result in an increased application of imaging‐based data to guide the development of mathematical models for quantitative investigation of macromolecules.

## CONFLICT OF INTEREST

The authors have declared no conflicts of interest for this article.

## AUTHOR CONTRIBUTIONS

**Prashant Dogra:** Conceptualization; investigation; writing‐original draft; writing‐review and editing. **Joseph Butner:** Investigation; writing‐original draft; writing‐review and editing. **Sara Nizzero:** Writing‐original draft; writing‐review and editing. **Javier Ruiz Ramírez:** Writing‐original draft; writing‐review and editing. **Achraf Noureddine:** Writing‐original draft; writing‐review and editing. **María Peláez:** Writing‐original draft. **Dalia Elganainy:** Writing‐original draft. **Zhen Yang:** Writing‐original draft. **Anh‐Dung Le:** Writing‐original draft. **Shreya Goel:** Writing‐review and editing. **Hon Sing Leong:** Writing‐review and editing. **Eugene Koay:** Writing‐review and editing. **C. Jeffrey Brinker:** Writing‐review and editing. **Vittorio Cristini:** Writing‐review and editing.** Zhihui Wang:** Conceptualization; investigation; supervision; writing‐original draft; writing‐review and editing.

## RELATED WIREs ARTICLE

Engineering of radiolabeled iron oxide nanoparticles for dual‐modality imaging
